# Atlanta Society of Medicine

**Published:** 1890-03

**Authors:** L. P. Kennedy

**Affiliations:** Reporter


### ATLANTA SOCIETY OF MEDICINE.

Feb. 18, 1890.

Atlanta Society of Medicine met with Dr. F .W. McRae, Vice-President, in the chair, in the absence of Dr. W. P. Nicolson, President.

Dr. Hutchins reported a case of *acne vulgaris* affecting the back. Patient, a medical student, aged 28. Disease began on the face at the age of 16, continuing for nine years, and an occasional lesion still recurs. Began on chest ten years ago and still present in slight degree. Began on back at the age of 22. Diagnosis made by presence of comedones, red papules and the rapidity with which these went on to pustulation, as well as the fact that the comedo was often visible in the center of the lesion. The course of individual papules of syphilis would be much slower, the color would not be so bright a red—the lesions of acne pass through stages in about a week. A papulo-pustular or a pustular syphilide would scarcely occur in so robust a subject. Previous history of some assistance.

No general defect in health save tendency to constipation. Pigmented points present which result from inflammation, deposit of pigment from breaking down of inflammatory products. Has been treated for syphilis for a year, and also took treatment at Hot Springs. Actually gained forty pounds under specific treatment. Began present treatment with scrubbing back with "spiritus saponis kalinus" (two parts green soap, one part alcohol), followed with application of a one per cent. solution of bichloride of mercury in equal parts alcohol and water. Slight vesiculation was produced and the lotion was diluted one-half. After three weeks of treatment the recurrence of papules is very limited.

Dr. K. C. Divine presented a patient—male, aged 30, married—upon whom he operated for hydrocele, June, 1889, removing several ounces of fluid, and injecting a 75 per cent. solution of carbolic acid. The operation was repeated in a few weeks, much to the relief of the patient. But the case was presented, not that the hydrocele itself was of peculiar interest, but as complicating an enlargement of right leg, it being nearly twice the size of the left. Swelling extended the whole length of leg. Patient claims that his trouble has existed since he was two years old. Patient leads an active life; is fond of sport, often spending a day hunting without any ill effect, except, perhaps, a temporary increase in the swelling. No history of specific trouble; no history of injury. Thorough examination failed to reveal any tumor or growth. History of case excluded elephantiasis. Dr. G. H. Noble thought the cause was to be found in some obstruction to venous return. Dr. F. W. McRae concurred in the opinion just expressed.

### THREE CASES ILLUSTRATING THE NECESSITY OF VAGINAL EXAMINATION IN OBSCURE CASES OF REFLEX PAIN.

BY G. H. NOBLE, M. D., ATLANTA, GA.

We not infrequently meet with obscure cases pronounced incurable which, when traced to the true cause, prove simple in the extreme. They are examples of violation of one of the first principles of diagnosis; that is, the examination of every organ in the body.

As an illustration I shall select three out of quite a number of cases I have met with. The first was a resident of this city—a lady of bilious temperament and mother of eight children. She enjoyed good health all of her life except an intolerable itching of the parts supplied by the external nasal branch of the opthalmic nerve. The paroxysms would come several times a day and last for more than an hour each time. So distressing were the attacks that she was in fear of losing her mind. She had exhausted the skill of many reputable physicians and neurologists North and East, without any relief. She had received nearly as many explanations of the causes as she had physicians, except, perhaps, indigestion and catarrh of the bile ducts were in the lead. She was questioned closely and examined carefully except for disease of the pelvic organs—she having stated "that she felt no inconvenience and had no symptoms of uterine disease." But afterwards the fact was elicited that she had metrorrhagia, which she attributed to the change of life, and regarded as normal flushings. We discovered a bilateral laceration of the cervix, extending into the vaginal junction, subinvolution with considerable engorgement, and some other minor symptoms usually attending such conditions. It was then suggested to her that the itching might be a reflex disturbance dependent upon the condition of the uterus, and advised treatment and repair of the cervix, to which she readily assented.

Under treatment by the glycerine tampon the paroxysms grew less severe and frequent, until the day the laceration was closed, when they disappeared entirely. She has now passed a period of eight years without the slightest evidence of return of the disease.

The second case was a large fleshy lady from Chillocoatia, Mo. She was anæmic, and slightly chlorotic. She suffered from an intolerable dyspepsia for which she had received no relief from the many professional men she had consulted. She gave no history of uterine diseases, but as a last resort an examination was made with the hope of finding some explanation of the matter. We found about the same condition as in the above case and gave the same advice. She experienced relief at times during the treatment, but was not materially benefited until the cervix was closed. Then like magic the dyspepsia disappeared, and for two years remained well, since that time I have heard nothing from her and am unable to state her condition.

The third case was a lady from Indiana, æt. 35, married 13 years. Had 3 children. Her bad health began six months after last confinement, four years ago. She became very weak, but afterwards recovered and moved to Montgomery, Ala., where she had some malarial impressions, but again recovered and returned to her home in Indiana, but continued to have pains at the lower angle of the scapula and intercostal neuralgia on the left side, and many pains from the abdomen to the limbs. When located in the bowels she claims that it caused dysentery. She had no pain with menstruation nor other menstrual troubles. She was sometimes constipated (hard, lumpy actions), generally alternating with diarrhœa, which would last for days and even months. Had heavy, weighty sensation in the stomach, especially after eating. A change in diet seems to have had no effect upon the symptoms. Head felt heavy and stupid, and she claimed to have high fever at times. Pains were worse in the daytime, and had been losing flesh and strength steadily. The pains in the shoulders and intercostal neuralgia were constant.

The above history naturally directed my attention to constipation, and I expressed the opinion that it was the probable cause of the trouble. She stated, however, that the same opinion had been advanced before, and that attention to the constipation did not relieve her of the pains. However, I gave her a prescription for the condition of the bowels, also one of aconite for the neuralgia. I promised her nothing, as she refused a vaginal examination and I could not attribute the symptoms to disease of any other organs. She returned in two weeks unimproved and complained of a slight leucorrhœa. I made a vaginal examination and found a laceration on the right side of the cervix, extending externally to the vaginal junction, and internally a white cicatricial line reaching to the internal os; also, subinvolution, engorgement, endometritis and erosion of the os. I stopped all medication except the laxative, and resorted to the glycerine tampon, which afforded her slight relief. The day that the cervix was thoroughly dilated and the old scar softened and stretched the pain disappeared. At the end of a month, I left off the conical tampon and the pains returned. It was again resumed and the pains disappeared.

These cases fully illustrate the necessity of vaginal examinations in obscure cases when the cause of pain cannot be ascribed to disease in other parts of the body. Especially are we liable to be thrown off our guard in making a diagnosis when there are no symptoms of disease of the uterus and appendages.

Dr. V. O. Hardon said he wished to exphasize the remarks of Dr. Noble. Many otherwise obscure and intractable cases would yield readily to an operation on the cervix, it being lacerated, and so giving rise to the many reflex symptoms of which the patient complained. Dr. H. Huzza said he had seen an aggravated case of acne disappear after an operation on lacerated cervix, showing the acne to be symptomatic.

L. P. KENNEDY, Reporter.

